# Gut Microbiome Dysbiosis is Associated With Human T‐Lymphotropic Virus Type 1 (HTLV‐1) Infection and Disease Progression to HTLV‐1‐Associated Myelopathy/Tropical Spastic Paraparesis: A Cross‐Sectional Study

**DOI:** 10.1002/smmd.70024

**Published:** 2026-01-09

**Authors:** Lorena Abreu Fernandes, Ana Olivia de Souza, Youko Nukui, Rosa Maria Marcusso, Augusto César Penalva de Oliveira, Jorge Casseb, Patricia Bianca Clissa, Silas G. Villas‐Boas, Sabri Saeed Sanabani

**Affiliations:** ^1^ Postgraduate Program in Translational Medicine, Department of Medicine Federal University of São Paulo (UNIFESP) São Paulo Brazil; ^2^ Development and Innovation Laboratory Butantan Institute São Paulo Brazil; ^3^ Department of Hematology, Faculty of Medicine University of São Paulo São Paulo Brazil; ^4^ Department of Neurology Emilio Ribas Institute of Infectious Diseases São Paulo Brazil; ^5^ Laboratory of Medical Investigation LIM‐56, Division of Dermatology Faculty of Medicine, University of São Paulo São Paulo Brazil; ^6^ Immunopathology Laboratory Butantan Institute São Paulo Brazil; ^7^ Luxembourg Institute of Science and Technology Esch‐sur‐Alzette Luxembourg; ^8^ Laboratory of Medical Investigation LIM‐03, Division of Dermatology Faculty of Medicine, University of São Paulo São Paulo Brazil

## Abstract

Human T‐lymphotropic virus type 1 (HTLV‐1)‐associated myelopathy/tropical spastic paraparesis (HAM/TSP) is a chronic neuroinflammatory disease. Given the established role of the gut‐brain axis in other neurological diseases such as multiple sclerosis, the role of the gut microbiome in the pathogenesis of HAM/TSP remains a critical unexplored area. The aim of this study was to characterize alterations in the gut microbiome associated with HTLV‐1 infection and its clinical stages. We performed a cross‐sectional analysis of the gut microbiome from 112 Brazilian individuals, including 24 healthy controls and 88 HTLV‐1‐infected individuals at different disease stages: 38 HAM patients, 17 patients with intermediate syndromes, and 33 asymptomatic carriers. Fecal samples were collected and analyzed using Illumina MiSeq sequencing to assess bacterial composition and diversity. Functional analysis was performed to identify differentially enriched gene categories and Kyoto Encyclopedia of Genes and Genomes (KEGG) modules. Significant dysbiosis was observed in HTLV‐1‐infected individuals, characterized by reduced bacterial diversity, an inverted Firmicutes/Bacteroidetes ratio, and specific changes in bacterial genera. Notably, HAM patients exhibited decreased *Faecalibacterium* and increased *Ruminococcus_g2* abundance. These associations should be interpreted with caution, as patient cohorts were significantly older and differed in sex distribution from healthy controls. Functional analysis revealed 13 differentially enriched gene categories and five KEGG modules that were more abundant in HAM patients, indicating alterations in metabolic processes. These findings provide the first comprehensive insight into gut microbiome changes associated with HTLV‐1 infection and disease progression. This study provides the first comprehensive insight into gut microbiome changes associated with HTLV‐1 infection and disease progression. The identified microbial signatures and functional alterations highlight potential diagnostic and therapeutic targets for HTLV‐1‐associated diseases, particularly HAM. These findings open new avenues for further research and clinical applications.

## Key Points

- We provide the first comprehensive analysis of changes in the gut bacteriome at different clinical stages of HTLV‐1 infection.
- HTLV‐1 infection is associated with a dysbiosis that includes reduced bacterial diversity and an inverted Firmicutes/Bacteroidetes ratio.
- Disease progression to HAM is characterized by depletion of *Faecalibacterium* and enrichment of *Ruminococcus_g2*, reflecting microbial signatures of neuroinflammatory severity.
- Functional shifts in microbial metabolic pathways indicate potential biomarkers and offer hypotheses for future diagnostic and therapeutic research in HTLV‐1‐associated diseases.

## Graphical abstract

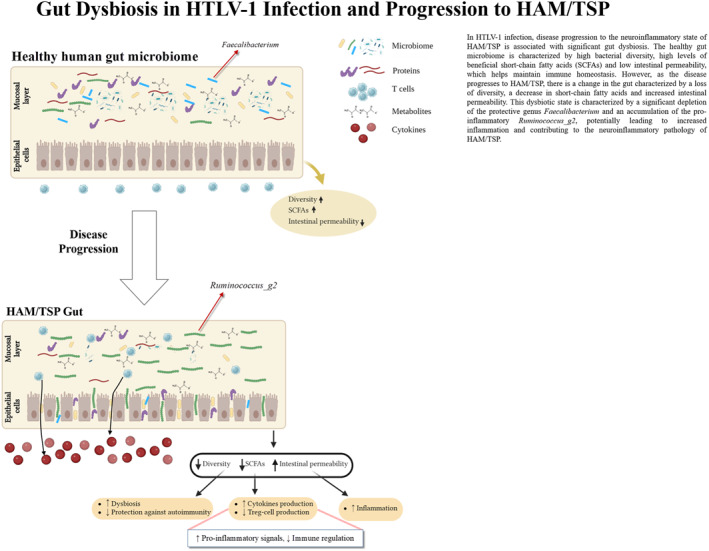

## 1 Introduction

Human T‐cell lymphotropic virus type 1 (HTLV‐1) is a retrovirus that affects an estimated 10–20 million people worldwide [1]. HTLV‐1 was first isolated from a patient with adult T‐cell leukemia/lymphoma (ATLL) and is disproportionately prevalent in regions such as southwestern Japan, the Caribbean, South America, and parts of central Africa [1]. The clinical manifestations of the virus range from asymptomatic infection to severe disease such as HTLV‐1‐associated myelopathy (HAM), which occurs in 1%–5% of people with HTLV‐1 (PLHTLV‐1) [1]. HAM is characterized by progressive lower limb weakness, spasticity and bladder or bowel dysfunction and is similar to the primary progressive form of multiple sclerosis [2].

The pathogenesis of HAM involves chronic immune activation against HTLV‐1 and infiltration of inflammatory cells into the central nervous system (CNS), which contributes to clinical disability [3]. Both host‐ and virus‐related factors are involved in the progression of the disease [4]. HTLV‐1 proviral load (PVL) is an important marker that is significantly elevated in peripheral blood mononuclear cells (PBMCs) and cerebrospinal fluid (CSF) of HAM patients compared with asymptomatic carriers [5]. However, the mechanisms responsible for the differences in clinical outcomes are not yet sufficiently understood. Early diagnosis of HAM is crucial for timely treatment of symptoms. However, there is no specific treatment for HAM, which emphasizes the need for novel diagnostic markers and therapeutic targets [6].

The human gut microbiome, the body's largest microbial ecosystem, plays a central role in maintaining health and influencing disease [7]. Recent research has highlighted the gut‐brain axis (GBA) as a bidirectional communication network linking the gut microbiota to neurological health [8]. The gut microbiota influences distant organs, including the brain, through immune modulation, metabolite production and signaling pathways [9]. Dysbiosis in the gut microbiota has been associated with immune‐related neurological diseases such as multiple sclerosis (MS) [10]. For example, fecal transplants from twins with MS in mice resulted in a higher rate of experimental autoimmune encephalomyelitis compared with transplants from healthy twins, which correlated with increased anti‐inflammatory interleukin‐10 (IL‐10) production [11, 12].

The biological similarities between HAM and MS suggest that alterations in the gut microbiota may play a role in the pathogenesis of HAM, a largely unexplored area. The present study attempts to fill this gap by characterizing the gut bacteriome at different clinical stages of HTLV‐1 infection using 16S rRNA sequencing. The cohort includes 38 HAM patients, 17 with intermediate syndromes (IS), 33 asymptomatic carriers (ASC) and 24 healthy controls (HC), all living in São Paulo, Brazil. IS represents a transitional clinical state between asymptomatic carriers and full‐blown myelopathy, characterized by inflammatory symptoms and high PVL, possibly marking an early development of HAM [13]. The aim of the study is to compare gut bacteriome profiles between PLHTLV‐1 and HC, to characterize bacteriome changes in different clinical stages of HTLV‐1 and to identify microbial markers associated with the progression of HAM.

As a crucial first step toward understanding this possible link, this study performed the first comprehensive characterization of the gut bacteriome at different clinical stages of HTLV‐1 infection. Our aim was not to establish a causal link but to identify consistent microbial signatures and functional changes associated with the presence and progression of the disease. By mapping this unexplored landscape, our study aims to generate key hypotheses and provide insights that could pave the way for microbial markers for earlier diagnosis and inform therapeutic strategies targeting the gut microbiome.

## 2 Materials and Methods

### 2.1 Ethics Statement

This study was approved by the Institutional Review Board of the Emilio Ribas Institute (CAAE: 68008923.5.1001.0061) and the Hospital das Clínicas (CAAE: 65467022.7.0000.0068). Written informed consent was obtained from all participants, and demographic and clinical data were collected using questionnaires and hospital electronic medical records.

### 2.2 Subjects

Clinical classification of HTLV‐1 patients was based on the World Health Organization criteria, while patients with IS were identified using the Emilio‐Ribas criteria [14]. Exclusion criteria included recent use of antibiotics or probiotics (within 2 weeks), coexisting diseases or tumors, and incomplete clinical data. The control group consisted of 24 healthy family members of patients who were selected based on their presumably similar dietary habits to minimize differences in bacteriome composition. The control group had to be tested negative for HTLV‐1, not have any diagnosed diseases and not take antibiotics or probiotics for at least 4 weeks prior to sampling.

### 2.3 Sample Collection

Participants collected their stool samples using Norgen Stool Nucleic Acid Collection and Preservation Tubes (Norgen Biotek) and received detailed instructions, including a video, to ensure contamination‐free handling. The samples were stored at room temperature until they arrived in the laboratory, according to the manufacturer's guidelines.

### 2.4 DNA Extraction and Library Preparations

DNA was extracted from up to 400 μL of stool using the Norgen Stool DNA Isolation Kit, with quality and concentration determined by agarose gel electrophoresis and a Qubit 2.0 fluorometer (Life Technologies). The extracted DNA was stored at −20°C until further use.

Library preparation involved PCR amplification of the variable region V3–V4 of the 16S rRNA gene with Bact_341F/Bact_805R primers as previously described [15]. Purified amplicons were indexed in a second PCR, pooled in equimolar amounts, diluted to 4 nM and sequenced on an Illumina MiSeq platform (300 bp paired‐end).

Processing of raw reads began with quality filtering (< Q25) using Trimmomatic v0.32 [16]. Paired‐end sequences were merged with VSEARCH v2.13.4 [17], and primers were trimmed using the alignment algorithm of Miller and Myers [18] with a similarity cutoff of 0.8. Non‐specific amplicons were identified using nhmmer (HMMER v3.2.1) [19]. Unique reads were dereplicated and clustered into OTUs using the EzBioCloud 16S rRNA database [20] via VSEARCH. Chimeric sequences were removed with UCHIME [21], and singletons were excluded. Data were normalized by rarefaction to the least sequenced sample size.

### 2.5 Gut Bacteriome Profiling and Diversity

Given the exploratory nature of this study and the inherent complexity of gut microbiome analysis, our initial characterization focused on taxonomic shifts at the phylum and genus level, which are commonly used to identify broad community changes. The gut bacterial composition from fecal samples, based on 16S rRNA sequencing, was analyzed using the EzBioCloud platform at both the phylum and genus levels. Alpha diversity indices (ACE, Chao1, Shannon, Simpson, and phylogenetic diversity) and beta diversity measures (Jensen‐Shannon divergence and Bray‐Curtis dissimilarity) were calculated, with beta diversity differences assessed using PERMANOVA. All the above analysis was performed in EzBioCloud 16S‐based Microbiome Taxonomic Profiling (MTP), a bioinformatics cloud platform from ChunLab that automatically processes the uploaded fastq data, which are converted into MTP data units. An MTP represents a single metagenomic or microbiome sample.

### 2.6 qRT‐PCR Validation of OTUs

Based on statistical significance and LDA scores exceeding 2.0, two OTUs were identified as potential markers for HAM/TSP and selected for qRT‐PCR validation. DNA previously extracted from the study cohort, including 38 HAM/TSP patients and 33 asymptomatic carriers (ASC), was used for validation analysis. The intermediate syndromes (IS) group was excluded from the validation process because they did not show significant differences in microbial composition compared with HAM patients during initial analyses. To explore potential species‐level differences within significantly altered genera, we selected Faecalibacterium prausnitzii and Ruminococcus bromii. *Faecalibacterium prausnitzii* was selected as the most clinically relevant anti‐inflammatory species within its genus, while *R. bromii* was chosen as a representative of the significantly altered *Ruminococcus* genus, both of which showed differential abundance in our sequencing data. This selection aimed to provide initial insights into species‐level variations.

The abundance of two differentially regulated genera, *F. prausnitzii* (*F*. *prausnitzii*) and *R. bromii*, was assessed using specific primers: *F*. *prausnitzii* (in‐house‐designed primers FpF: 5′‐CCATGAATTGCCTTCAAAACTGTT‐3′ and FpR: 5′‐GAGCCTCAGCGTCAGTTGGT‐3′) and *R*. *bromii* (RbF: 5′‐TCGCGGATCAGAATGCCGCGG‐3′ and RbR: 5′‐CCTCACGAGGTTGGACTACTGA‐3′). For normalization, the 16S rRNA gene was amplified using the universal primers 16S‐F: 5′‐CGGTGAATACGTTCYCGG‐3′ and 16S‐R: 5′‐GGWTACCTTGTTACGACTT‐3′.

Amplification was performed on a Thermo Fisher Scientific 7500 Real‐Time PCR System using SsoAdvanced Universal SYBR Green Supermix (Bio‐Rad). Each 10 μL reaction mixture contained 0.25 μL of each primer, 5 μL SYBR Green Supermix, 2.5 μL nuclease‐free water, and 2 μL template DNA. The thermal cycling protocol included an initial denaturation step at 98°C for 3 min, followed by 35 cycles of denaturation at 98°C for 10 s and annealing/extension at 55°C for 30 s. All reactions were performed in duplicate, and relative quantification was calculated using the 2^−ΔΔCT^ method to compare the abundance of target genera between HAM patients and ASC.

### 2.7 Statistical Analysis

Continuous variables were presented in the form of mean values (standard deviations) or median values (interquartile ranges). Differences between subjects in the HTLV‐1‐infected group (HAM = 38, IS = 17 and ASCs = 33) and healthy controls (*n* = 24) were compared using Student's *t*‐test for normal continuous variables and Wilcoxon rank sum test for non‐normal continuous variables. Statistical analyzes were performed using the R package implemented in BioRender (https://app.biorender.com/). Statistical significance was defined by *p* < 0.05 (two‐tailed test). When multiple comparisons were performed, *p*‐values were adjusted for false discovery rate (FDR) using the Benjamini‐Hochberg method. The data were not transformed, normalized, or examined for outliers in the study.

## 3 Results

### 3.1 Study Cohort

A prospective study with rigorous diagnostic and exclusion procedures was conducted at the Emilio Ribas Institute of Infectious Diseases and Hospital das Clinicas in São Paulo, Brazil. A total of 113 stool samples were collected from outpatient clinics, 112 of which were successfully included in the analysis. The study cohort included 24 healthy controls and 88 PLHTLV‐1. The PLHTLV‐1 were divided into three clinical categories: 38 patients with HAM, 17 with IS and 33 with ASC. The gut bacteriome was characterized in all four groups, allowing a comprehensive analysis of the bacteriome in both healthy individuals and those at different stages of HTLV‐1 infection. An attempt was made to assign participants to the PLHTLV‐1 group by age, but a balanced gender distribution proved difficult due to the higher prevalence of the disease in women. Of note, constipation was significantly more common in PHLV‐1 patients than in HC patients, with HAM patients having the highest incidence (Table 1). Fecal incontinence was found in two HAM patients, but not in the other groups, further emphasizing the different clinical manifestations of this subgroup.

**TABLE 1:** Characteristics of the subjects.

Variable	HAM patients (*n* = 38)	IS patients (*n* = 17)	ASC (*n* = 33)	HCs (*n* = 24)	*p* value
Age (year), mean ± SD	54.9 ± 13.4	56 ± 9.5	58.3 ± 13.3	38.6 ± 15.2	< 0.0001
Sex, *n* (%)					0.049
Male	11 (28.9)	2 (11.8)	8 (24.4)	12 (50.0)	
Female	27 (71.1)	15 (88.2)	25 (75.6)	12 (50.0)	
Constipation, *n* (%)	30 (78.9)	6 (35.3)	10 (30.3)	5 (20.8)	< 0.00001
Fecal incontinence, *n* (%)	2 (5.3)	0 (0.0)	0 (0.0)	0 (0.0)	

Abbreviations: ASC, asymptomatic carriers; HAM, HTLV‐1‐associated myelopathy; HCs, healthy controls; IS, intermediate syndromes.

### 3.2 Characterization of the Gut Bacteriome in PLHTLV‐1 and HCs

**FIGURE 1:**
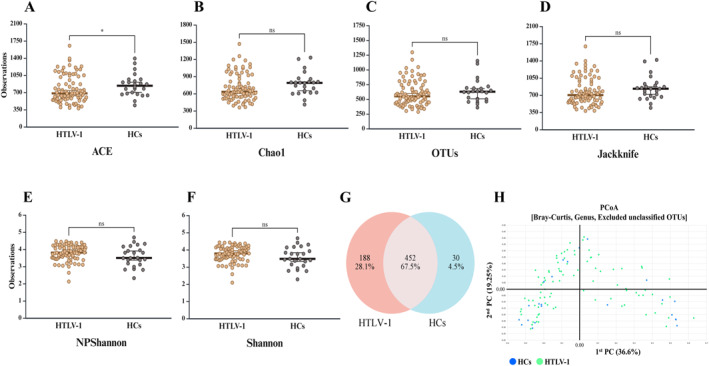
Alpha and beta diversity comparisons of the gut microbiome between PLHTLV‐1 and healthy controls. (A) Ace index, (B) Chao1 index, (C) OTU counts, and (D) Jackknife index show a trend toward lower bacterial diversity in PLHTLV‐1 compared with HCs, with a significant difference only in the Ace index (*p < 0.05). (E) NPShannon index and (F) Shannon index show no significant differences between groups. (G) Venn diagram showing overlap of OTUs between groups: 452 of 670 OTUs are shared, while 188 are unique to PLHTLV‐1 (*n* = 88) and 30 are unique to HCs (*n* = 24). (H) PCoA plot based on Bray–Curtis dissimilarity (genus level, excluding unclassified OTUs) shows no distinct clustering between PLHTLV‐1 and HCs.

First, to assess the overall impact of HTLV‐1 infection on the gut microbial community, we compared the PLHTLV‐1 (*n* = 88) and HC (*n* = 24) groups. It is critical to first note that these groups differed significantly in age and sex (Table 1). The patient cohort was notably older, an inherent challenge in studying a disease like HAM with a long latency period before symptom onset. While our statistical approach does not adjust for these demographic variables, our recruitment of healthy family members as controls was a deliberate strategy to minimize the powerful confounding effects of diet and household environment. The following results should be interpreted within this context. Our analysis of community Alpha diversity revealed a trend toward lower diversity in the PLHTLV‐1 group (Figure 1). Specifically, the ACE index revealed a statistically significant reduction in richness (*p* < 0.01, Figure 1A), while other richness estimators like Chao1, OTUs, and Jackknife showed a similar non‐significant trend (Figure 1B–D). Indices measuring evenness, such as NP‐Shannon and Shannon, showed no significant differences between the groups (Figure 1E,F). Beta diversity analysis, used to compare the overall microbial composition between groups, was visualized with PCoA plots. These plots showed no distinct clustering that differentiated the PLHTLV‐1 bacteriome from HCs at the OTU level, suggesting that the overall community structures, while different, are not dramatically altered (Figure 1H). A Venn diagram showed a total of 670 OTUs, with 452 (67.5%) being common to both groups (Figure 1G).

**FIGURE 2:**
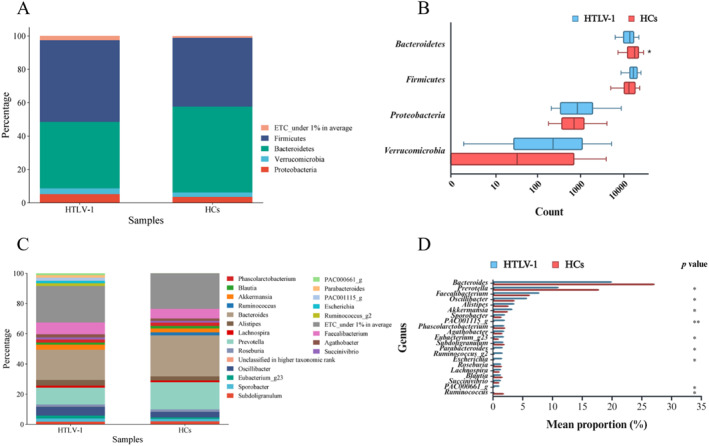
Taxonomic composition and relative abundance of the gut bacteriome in the PLHTLV‐1 group (*n* = 88) and healthy controls (HCs, *n* = 24). (A) Average relative abundance of bacterial communities at the phylum level. (B) Among phyla, only Bacteroidetes were significantly reduced in the PLHTLV‐1 group compared with HCs. (C) Average relative abundance of bacterial communities at the genus level. (D) Six genera were significantly enriched, while two were significantly reduced in the PLHTLV‐1 group compared with HCs.

Having established these subtle but significant differences in community diversity, we next investigated the specific taxonomic alterations driving this dysbiosis (Figure 2). We analyzed the taxonomic composition and alterations of the gut bacteriome in the whole cohort (*n* = 112). The average composition and relative abundance of the bacterial community in PLHTLV‐1 and HC at the phylum and genus level are shown in Figure 2A,C, respectively. Of the four main phyla (Firmicutes, Bacteroidetes, Proteobacteria, and Verrucomicrobia), only the Bacteroidetes were significantly reduced in PLHTLV‐1 compared with HC (Wilcoxon rank sum test, *p* = 0.012, Figure 2B). At the genus level, of the 21 genera detected, six were significantly enriched, while *Prevotella* and *Ruminococcus* were significantly reduced in PLHTLV‐1 compared with HC (all *p* < 0.05, Figure 2D). Further comparisons of the bacterial composition of the gut bacteriome between groups at the class, order and family level are shown in Figures [Link], [Link], [Link], [Link], [Link], [Link], [Link], [Link], [Link]. Of note, at the class level, Verrucomicrobiae were significantly enriched while Bacteroidia were reduced in PLHTLV‐1 (*p* < 0.05). At the order level, only the Bacteroidales were significantly reduced (*p* < 0.05), and at the family level, four bacterial populations, including Ruminococcaceae and Rikenellaceae, were significantly enriched in PLHTLV‐1 (all *p* < 0.05).

Heatmap analysis of 17 major OTUs (Figure S10) revealed subtle differences in bacterial abundance between groups. While certain genera were differentially abundant in the different samples, the dendrogram in the upper part of the heatmap showed mixed clustering in both PLHTLV‐1 and HC samples. This pattern suggests that the overall structures of the bacterial community are similar between the two groups and differ only slightly. The lack of pronounced clustering suggests that HTLV‐1 infection does not dramatically alter the composition of the gut microbiome at the OTU level.

**FIGURE 3:**
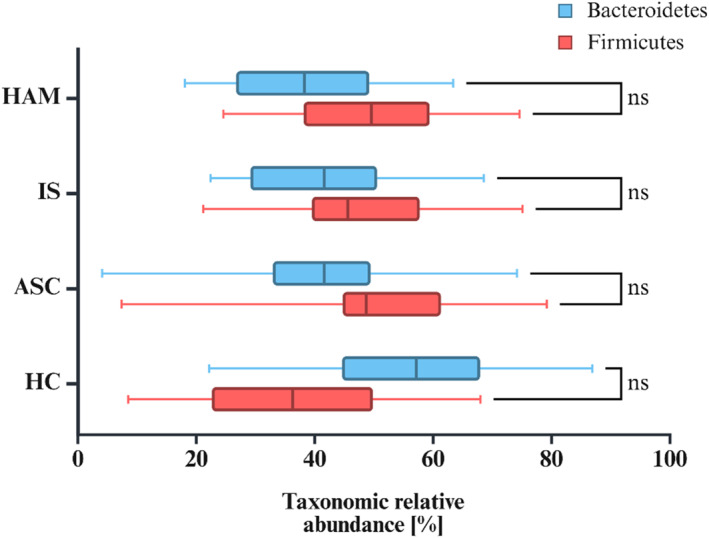
Relative abundance of Bacteroidetes and Firmicutes in PLHTLV‐1 subgroups (HAM, IS, ASC) and healthy controls (HCs). In HCs, Bacteroidetes tended to be more abundant, whereas in PLHTLV‐1 groups, Firmicutes tended to be more abundant, suggesting a reversal of the Firmicutes/Bacteroidetes ratio. However, these differences were not statistically significant (ns).

Next, we focused on the Firmicutes to Bacteroidetes (F/B) ratio, as it is a widely recognized indicator of gut bacterial health and balance. Considering the immune‐mediated nature of HTLV‐1‐associated diseases, we hypothesized that this ratio might shed light on the relationship between HTLV‐1 infection and the composition of the gut bacteriome. As shown in Figure 3, our analysis revealed a striking difference in the F/B ratio between the HC‐ and HTLV‐1‐infected groups. In the HC group, Bacteroidetes predominated over Firmicutes. In contrast, all HTLV‐1‐infected groups (HAM, IS and ASC) showed an inverse ratio, with Firmicutes dominating over Bacteroidetes. This pattern was the same in all stages of HTLV‐1 infection, from ASC to individuals with HAM. Other bacterial phyla, including Proteobacteria, Actinobacteria and Verrucomicrobia, showed relatively minor differences in all groups.

### 3.3 Alterations of the Gut Bacteriome in PLHTLV‐1

**FIGURE 4:**
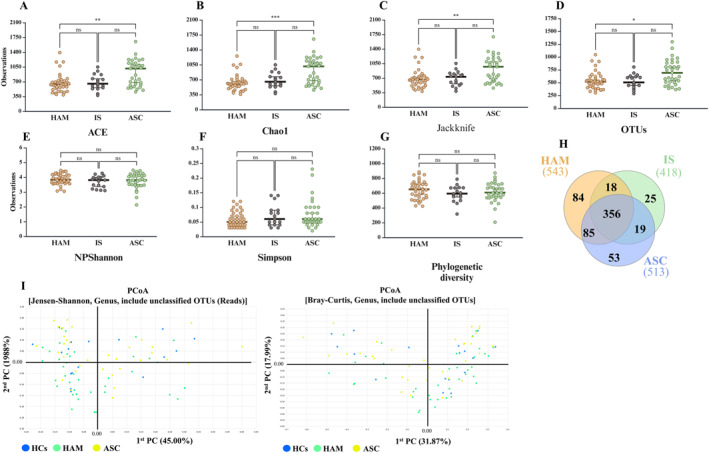
Reduced alpha diversity and distinct beta diversity patterns in the gut microbiome of the HAM group. The estimates of (A) Ace index, (B) Chao1 index, (C) Jackknife index, and (D) OTU counts show that bacterial diversity in the gut of HAM patients is significantly lower compared with ASCs (*p < 0.05; **p < 0.01; ***p < 0.001). (E) NPShannon index, (F) Simpson index, and (G) phylogenetic diversity index show no significant differences between the three groups. (H) Venn diagram showing the overlap between groups: 356 of the total 1474 OTUs are common to all three groups, while 84 are found only in HAM patients (*n* = 38). (I) PCoA plots based on Jensen–Shannon and Bray–Curtis indices (genus level, including unclassified OTUs) show that HAM samples (green) are more broadly distributed, indicating greater variability.

To investigate alterations in the gut bacteriome across different clinical stages of PLHTLV‐1, we analyzed 88 stool samples divided into three groups based on clinical symptoms: 38 from HAM patients, 17 from IS patients, and 33 from ASC individuals. We first assessed α‐diversity using several richness and evenness indices. Species richness, as estimated by ACE, Chao, jackknife, and observed OTUs, was significantly reduced in HAM patients compared with ASC, but not in the IS group (Figure 4A–D). In contrast, NPShannon, Shannon, Simpson, and phylogenetic diversity indices showed no significant differences among the three groups (Figure 4E–G). A Venn diagram revealed a total of 640 OTUs, of which 356 were shared across all groups; 84 OTUs, 25 OTUs, and 53 OTUs were unique to HAM, IS, and ASC, respectively (Figure 4H). β‐diversity was examined by principal coordinate analysis (PCoA) using Jensen–Shannon and Bray–Curtis distances. In the Jensen–Shannon analysis, PC1 and PC2 accounted for 45.0% and 19.9% of the variance, respectively; in the Bray–Curtis analysis, they accounted for 31.9% and 18.0%, respectively (Figure 4I). HAM samples (green) appeared more widely distributed, IS samples (blue) were more tightly clustered, and ASC samples (yellow) showed an intermediate pattern. PERMANOVA confirmed significant separation between HAM and ASC (Jensen–Shannon, *p* = 0.02; Bray–Curtis, *p* = 0.008) (Figure S11A–F). Overall, diversity metrics indicated subtle but significant differences in community structure, particularly between HAM and ASC.

**FIGURE 5:**
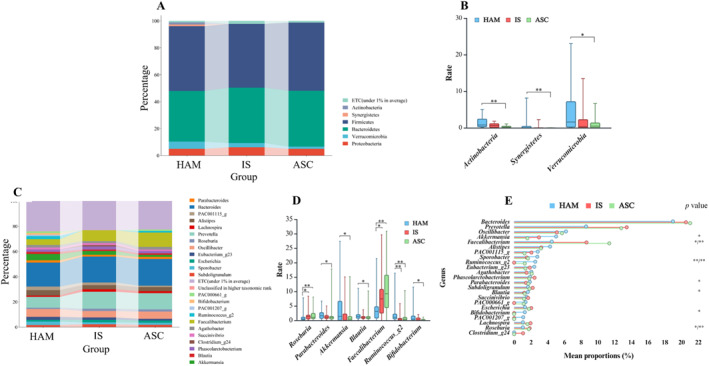
Taxonomic composition and relative abundance of the gut bacteriome between HAM (*n* = 38), IS (*n* = 17), and ASC groups (*n* = 33). (A) Average composition and relative abundance of the bacterial community in each group at the phylum level. (B) Compared to ASC patients, Verrucomicrobia, Synergistetes and Actinobacteria were significantly increased in HAM patients. (C) Average composition and relative abundance of the bacterial community in the three groups at genus level. (D) Five genera were significantly enriched, while two genera significantly decreased with the progression of HAM in the three groups. (E) Three genera were significantly enriched in HAM patients compared to ASC, while four genera were significantly reduced in HAM patients, but not compared to the IS group. *p < 0.05; **p < 0.01.

We then examined the taxonomic profiles of the gut bacteriome. At the phylum level, Verrucomicrobia, Synergistetes, and Actinobacteria were significantly increased in HAM patients compared with ASC (Figure 5A,B). No significant differences were observed between HAM and IS or between IS and ASC. At the genus level, five genera, including *Parabacteroides*, *Akkermansia*, and *Blautia*, were significantly enriched, whereas *Roseburia* and *Faecalibacterium* were significantly reduced in HAM compared with ASC (Figure 5C,D). Among the 23 major genera detected, three were enriched and four were reduced in HAM versus ASC, but no significant changes were seen compared to IS (Figure 5E). The observed shifts in both diversity and composition suggest a disruption of microbial community balance with the progression to HAM, marked by reduced species richness and enrichment of certain taxa with potential inflammatory roles.

### 3.4 Crucial Bacteria in Connection With HAM

To find functional markers, we used linear discriminant analysis (LDA) with effect size analysis (LEfSe) in the KEGG databases. The LDA model identifies differentially abundant taxa between groups and estimates the effect size of each significantly different taxon [22]. We assessed the LEfSe of the three groups with a standard LDA score of ≥ 3.65 and *p* < 0.05 to search for a statistically significant biomarker at the genus level (Table 2). LEfSe analysis of all genera revealed 32 bacterial taxa with significant differences. LEfSe analysis revealed that the genus *Faecalibacterium* was most abundant in the ASC group, while *Ruminococcus_g2* was most abundant in the HAM group.

**TABLE 2:** LEfSe analysis of taxonomic biomarkers of gut microbiota among the HAM, IS, and ASC groups.

Taxon name	Taxon rank	LDA effect size	HAM	IS	ASC	*p*‐value
*Faecalibacterium*	Genus	4.56	4.50	8.65	11.38	0.0001
*Faecalibacterium prausnitzii group*	Species	4.50	4.41	8.41	10.54	0.0003
Verrucomicrobia	Phylum	4.27	5.33	3.11	1.64	0.0291
*Ruminococcus_g2*	Genus	3.94	2.53	0.71	1.27	0.0006
Synergistetes	Phylum	3.76	1.18	0.55	0.01	0.0051
Synergistales	Order	3.76	1.18	0.55	0.01	0.0051
Synergistia	Class	3.76	1.18	0.55	0.01	0.0051
Synergistaceae	Family	3.76	1.18	0.55	0.01	0.0051
AC160630_f	Family	3.76	1.05	0.00	0.15	0.0007
*Roseburia*	Genus	3.67	1.04	1.86	1.75	0.0085

### 3.5 Different KEGG Genes, Modules and Pathways of Gut Bacteria

A total of 69 KEGG orthologs (KO) were identified in the gut bacteriome dataset. Using the LEfSe algorithm, 13 KO terms with a LAD score > 2 and a *p*‐value < 0.05 were identified as differentially enriched (Table 3). In particular, functional gene categories coding for myo‐inositol‐2‐dehydrogenase/D‐chiro‐inositol‐1‐dehydrogenase were significantly overrepresented in HAM patients.

**TABLE 3:** The 13 most differentially enriched KEGG Orthology (KO) terms in the gut bacteriome were identified with a LAD score > 2 and *p*‐value < 0.05 using the LEfSe algorithm.

Ortholog	Definition	Pathway	Module	LDA effect size	*p*‐value	HAM	IS	ASC
K06400	Site‐specific DNA recombinase			2.58	0.002	0.137	0.199	0.212
K03327	Multidrug resistance protein, MATE family			2.41	0.009	0.262	0.319	0.312
K06147	ATP‐binding cassette, subfamily B, bacterial			2.39	0.005	0.159	0.198	0.207
K02315	DNA replication protein DnaC			2.19	0.001	0.071	0.100	0.102
K06919	Putative DNA primase/helicase			2.18	0.000	0.092	0.115	0.122
K03205	Type IV secretion system protein VirD4	ko03070	M00333	2.16	0.008	0.071	0.093	0.100
K03603	GntR family transcriptional regulator, negative regulator for fad regulon and positive regulator of fabA			2.14	0.000	0.025	0.044	0.052
K21471	Peptidoglycan DL‐endopeptidase CwlO			2.12	0.011	0.116	0.145	0.145
K03169	DNA topoisomerase III			2.11	0.001	0.083	0.108	0.111
K03496	Chromosome partitioning protein			2.10	0.037	0.139	0.170	0.168
K00010	Myo‐inositol 2‐dehydrogenase/D‐chiro‐inositol 1‐dehydrogenase	ko00521, ko00562, ko01100, ko01120, ko01130		2.07	0.017	0.105	0.080	0.082
K03086	RNA polymerase primary sigma factor			2.02	0.006	0.073	0.091	0.094
K08168	MFS transporter, DHA2 family, metal‐tetracycline‐proton antiporter		M00704	2.02	0.002	0.031	0.046	0.052

To understand the biological significance of these differences in the frequency of functional gene categories, all KO terms found were assigned to KEGG modules and pathway levels. Five different KEGG modules were identified, all of which were more frequent in HAM patients compared to IS and ASC groups (Table 4a). At the metabolic pathway level, using the same cut‐off criteria, only the pyrimidine pathway (ko00240) was identified as significantly different. To verify the profiles of the different metabolic pathways and modules, the MinPath inference method was used to compare the functional metagenomes of the three sample groups. Two additional metabolic pathways, cysteine and methionine metabolism (ko00270) and biofilm formation—*Pseudomonas aeruginosa* (ko02025), were calculated (Table 4b). The results also showed four other KEGG modules, two of which (M00609 and M00745) represent pathways for cysteine biosynthesis and amino acid degradation, respectively (Table 4c). The fact that different tools predicted different modules may indicate functional redundancy in the bacteriome, where multiple metabolic pathways may perform similar tasks. Discrepancies in the predictions of PICRUSt and MinPath are expected as these tools use different algorithms and databases, emphasizing the importance of using multiple tools for functional prediction in microbiome studies.

**TABLE 4a:** Metagenomic functional modules identified by PICRUSt analysis showed significant differences between the HAM, IS, and ASC groups.

Module	Definition	Pathway	LDA effect size	*p*‐value	HAM	IS	ASC
M00331	Type II general secretion pathway	ko03070	2.63	0.034	0.294	0.206	0.227
M00332	Type III secretion system	ko03070	2.54	0.049	0.264	0.201	0.205
M00099	Sphingosine biosynthesis	ko00600, ko01100	2.51	0.027	0.234	0.178	0.177
M00121	Heme biosynthesis, glutamate => heme	ko00860, ko01100, ko01110	2.48	0.007	0.414	0.377	0.362
M00567	Methanogenesis, CO_2_ => methane	ko00680, ko01100, ko01120, ko01200	2.46	0.046	0.288	0.237	0.239

**TABLE 4b:** Additional metagenomic functional modules identified by MinPath analysis showed significant differences between the HAM, IS, and ASC groups.

Module	Definition	Pathway	LDA effect size	*p*‐value	HAM	IS	ASC
M00609	Cysteine biosynthesis, methionine => cysteine	ko00270, ko01100, ko01230	2.60	0.007	0.187	0.252	0.258
M00081	Pectin degradation	ko00040, ko01100	2.55	0.007	0.252	0.300	0.313
M00519	YesM‐YesN two‐component regulatory system	ko02020	2.52	0.026	0.269	0.333	0.328
M00745	Imipenem resistance, repression of porin OprD		2.52	0.002	0.171	0.231	0.233

**TABLE 4c:** Differential KEGG pathways identified by MinPath analysis showed significant differences between the HAM, IS, and ASC groups.

Pathway	Definition	Module	Orthology	LDA effect size	*p*‐value	HAM	IS	ASC
ko00270	Cysteine and methionine metabolism	M00021, M00368, M00035, M00034, M00338, M00017, M00609	K01251	2.90	0.009	0.693	0.824	0.842
ko02025	Biofilm formation—*Pseudomonas aeruginosa*	M00820		2.89	0.045	0.565	0.699	0.706

#### 3.5.1 qRT‐PCR Validation Results

Our subsequent qRT‐PCR validation for the relative abundance of *F. prausnitzii* and *R. bromii* did not yield statistically significant differences between HAM patients and ASC (data not shown). Both species showed comparable levels of abundance in the two groups, as quantified using the 2^−ΔΔCT^ method.

## 4 Discussion

Disruption of the normal gut microbiome is increasingly recognized as a significant factor in the development and progression of chronic diseases, yet its role in HTLV‐1 infection remains unexplored [23, 24, 25, 26, 27, 28]. Our study addresses this critical gap by providing the first comprehensive investigation of the gut bacteriome across the clinical spectrum of HTLV‐1 infection. Using 16S rRNA sequencing in a cohort of 112 Brazilian participants, we discovered a significant dysbiosis associated with disease state, characterized by reduced bacterial diversity, a starkly inverted Firmicutes/Bacteroidetes ratio, and a substantial reshaping of the microbial community at the genus level. Most notably, progression to HTLV‐1‐associated HAM was associated with a significant decrease in the anti‐inflammatory genus *Faecalibacterium* and an increase in *Ruminococcus_g2*, highlighting key microbial shifts that correlate with disease severity.

### 4.1 Bacteriota Perturbations in HTLV‐1 Infection

Our analysis revealed significant differences in the gut bacteriome between the PLHTLV‐1 and HC groups, starting with overall community diversity. The significant difference in the ACE index, despite non‐significant differences in other richness indices, suggests that HTLV‐1 infection is associated with a reduction in microbial richness, particularly affecting less abundant species. Reduced microbial diversity is a common feature in various diseases such as obesity and inflammatory bowel disease and may indicate a less resilient gut ecosystem in HTLV‐1‐infected individuals [29, 30, 31]. However, the lack of significant differences in the NP‐Shannon and Shannon indices suggests that while species richness is reduced, the evenness of the community's distribution may be maintained. Furthermore, when comparing the overall community structures, the lack of clear clustering in the PCoA plots and the substantial overlap of OTUs between groups (67.5% shared) indicate that HTLV‐1 infection causes subtle but important shifts in the bacteriome rather than a complete and dramatic restructuring.

Having established these subtle but significant differences in community structure, we then investigated the specific taxonomic changes responsible. Perhaps the most striking of these is the reversal of the F/B ratio in all HTLV‐1‐infected groups compared with HCs. The F/B ratio has been widely discussed as a potential biomarker of gut health, with a higher ratio frequently reported in the context of an aging microbiome, obesity, and metabolic syndrome [32]. However, it is important to acknowledge that this is not a universally accepted paradigm, as an increased ratio is not consistently found across all diseases associated with a dysregulated microbiome [33]. Despite this ongoing debate, the consistent and stark reversal we observed across all stages of HTLV‐1 infection, from asymptomatic carriers to individuals with HAM, suggests a fundamental and robust alteration of the gut microbiome associated with the virus, rather than a finding that can be easily dismissed. This reinforces the idea that the overall functional balance of the microbial community, rather than a single biomarker or cluster, is likely what is altered in HTLV‐1 infection and contributes to its pathogenesis.

A key clinical factor that may influence this altered taxonomic landscape is constipation. Our study found important differences in clinical characteristics between the groups, most notably the significantly higher incidence of constipation in our HTLV‐1‐infected cohort, particularly in HAM patients (Table 1). This finding is consistent with previous reports and is an important clinical indicator [34]. Neurogenic bowel dysfunction is a known complication of HAM, and the altered intestinal transit time associated with constipation can strongly influence the composition of the gut microbiome [35]. Therefore, the dysbiosis we observed may be both a consequence of and contribute to the neurological symptoms that manifest as constipation in these patients [36, 37].

### 4.2 Microbial Signatures of Disease Progression to HAM/TSP

While the overall comparison between infected and healthy individuals is informative, the most critical insights emerge when analyzing the microbial shifts that correlate with disease progression from asymptomatic carriage to HAM/TSP. In terms of parallels with other viral and neurological diseases, the observed reduction in alpha diversity (species richness) in HAM patients is consistent with findings in HIV infections, another retroviral disease [38, 39]. The enrichment of certain bacterial genera such as *Akkermansia* and *Blautia* in HAM patients is also interesting, as these genera have been associated with other neurological diseases [40]. The decrease in *Faecalibacterium* and *Roseburia* in HAM patients is also noteworthy, as these genera are often associated with anti‐inflammatory properties [41]. Also, of interest is the increase in the genus *Verrucomicrobium* in HAM patients, which has been associated with neuroinflammatory processes in studies of other diseases [42]. Furthermore, the significant differences in PCoA plots, particularly between HAM and ASC groups, suggest that the progression of HAM is associated with significant, albeit subtle and complex, changes in the gut bacteriome.

It is important to note that while these parallels are informative, the specific context of HTLV‐1 infection may contribute to unique changes. The lack of significant differences between the IS group and other groups in this study highlights the complexity of the relationship between bacteriome changes and disease progression. Taxonomic analysis of biomarkers revealed that the genus *Faecalibacterium* was most abundant in the ASC group, while *Ruminococcus_g2* dominated in the HAM group. The significant decrease in *Faecalibacterium* in HAM patients could have a profound impact on disease progression and symptom severity. *Faecalibacterium*, particularly *F. prausnitzii*, are considered important players in the maintenance of intestinal homeostasis and are associated with various health benefits [43]. Its potential role as a key taxon in stabilizing the gut microbiota [44, 45] suggests that its depletion in HAM patients could contribute to increased microbial dysbiosis and gut instability. This genus is known for its strong anti‐inflammatory properties, in particular through the production of butyrate, a short‐chain fatty acid that is crucial for colonocyte health and the overall integrity of the intestinal barrier [43, 46]. The reduction of *Faecalibacterium* in HAM patients could contribute to increased intestinal inflammation and permeability, which could exacerbate the neuroinflammatory processes characteristic of the disease. This assumption is consistent with observations in other neurological disorders where lower *Faecalibacterium* concentrations have been associated with disease states, including Parkinson's disease, Alzheimer's disease, and clinical depression [47, 48, 49].

The dominance of *Ruminococcus_g2* in HAM patients represents a complex scenario for the dynamics of the gut bacteriome. While *Ruminococcus* species are known to produce beneficial SCFAs, their dominance may indicate a dysbiosis that could disrupt the delicate microbial balance that is critical for gut health [50]. The effects of this dominance on gut barrier function, immune responses and neuroinflammation are likely to be complex. SCFAs generally promote gut health, but their effects may be context‐dependent and influenced by concentration and the overall microbial community [51]. Paradoxically, some gut bacteria, including certain *Ruminococcus* strains, can exert pro‐inflammatory effects despite producing anti‐inflammatory metabolites such as butyrate [52]. The strain‐specific nature of the bacterial effects and the complex host‐microbe interactions associated with HTLV‐1 infection further complicate this relationship. The dominance of *Ruminococcus_g2* could reflect or cause metabolic shifts in the gut and indirectly influence inflammation and immune responses. Given these complexities, the specific role of *Ruminococcus_g2* in HAM pathology, whether protective, deleterious, or a consequence of disease processes, remains unclear, underscoring the need for further investigation.

An important result illustrating the complexity of microbial dysbiosis emerged from our attempt at species‐level validation. To investigate possible species‐level variation within these significantly altered genera, we selected *F. prausnitzii* and *R. bromii* for preliminary validation by qRT‐PCR. However, the qRT‐PCR results could not statistically confirm significant differences in their relative abundance between HAM patients and ASC. This lack of confirmation may be due to several factors, including the different sensitivity of sequencing compared to qRT‐PCR, potential variations in primer design, or the inherent complexity and variability within the gut microbial community that may not be fully captured by a limited number of validated species or a smaller validation cohort. Despite these challenges in species‐level validation, the robust genus‐level results from our comprehensive sequencing analysis remain critical and provide substantial insight into the ecological changes within the gut microbiome. The lack of species‐level confirmation does not invalidate the robust genus‐level results, but rather emphasizes that significant ecological changes at a higher taxonomic level are not always caused by a single, uniformly changing species. This emphasizes that our primary and most reliable conclusions from this 16S rRNA study are at the genus level.

### 4.3 Functional Consequences and Future Directions

Functional analysis using the PICRUSt and KEGG databases revealed 13 differentially enriched genes, with the myo‐inositol 2 dehydrogenase/D‐chiro‐inositol 1 dehydrogenase gene being overrepresented in HAM patients. The involvement of this gene in inositol metabolism suggests possible alterations in metabolic processes in HAM, and although similar observations have been made in other neuroinflammatory diseases [53], further studies are needed to establish a direct link in HAM. The identification of five KEGG modules more prevalent in HAM patients suggests specific functional changes in the gut bacteriome associated with the disease. Future studies could investigate whether these changes are comparable to those observed in other neurological disorders, such as HIV‐associated diseases [54]. The importance of the pyrimidine metabolic pathway (ko00240) in our analysis is particularly interesting. Pyrimidine metabolism plays a crucial role in nucleic acid synthesis and cellular energy production, and its increased activity may reflect the increased nucleotide requirement in HTLV‐1‐infected cells during chronic immune activation, potentially supporting T‐cell proliferation and contributing to inflammation.

While our findings show a strong association between gut dysbiosis and HAM, the cross‐sectional design of this study does not allow for the determination of causality. The observed changes, such as the depletion of *Faecalibacterium* and the enrichment of *Ruminococcus_g2*, represent robust biomarkers and are prime candidates for future research. Mechanistic studies, including the use of in vivo animal models and in vitro co‐culture systems, are now warranted to elucidate whether these microbial shifts are a cause or a consequence of HTLV‐1‐associated neuroinflammation.

We acknowledge several limitations in our study. First, its cross‐sectional design establishes association but cannot infer causation. While traditional, decades‐long longitudinal studies tracking individuals from initial HTLV‐1 infection are exceptionally challenging for a rare disease like HAM, a more focused approach could be employed. For instance, longitudinal analysis of individuals with IS would be valuable. Since these patients may represent an early stage of HAM development, following this group over time could provide crucial insights into the microbial shifts that precede or drive the transition to full‐blown myelopathy. Second, we acknowledge potential confounding factors. Most notably, there were significant differences in age and sex between our patient cohorts and the HC group (Table 1). The age disparity, in particular, is an inherent challenge in studying HAM/TSP, as the long latency period between HTLV‐1 infection and symptom onset means that patient groups are expected to be significantly older than an age‐matched healthy population. To mitigate other powerful confounders like diet and household environment, we deliberately recruited healthy family members as our HC group. Nevertheless, we could not account for all potential confounders, such as detailed dietary habits not captured by this design or the use of non‐antibiotic medications that might influence the gut microbiota. Third, this study characterizes the bacteriome, but does not explore the virome, mycobiome, or microbial metabolites (metabolome), which may also play a crucial role. Fourth, our study is based on sequencing of the 16S rRNA gene, which is suitable for genus‐level analysis but is known to be limited for definitive species‐level resolution. Our attempt at qRT‐PCR validation confirmed this complexity, and therefore, our conclusions focus on the significant changes observed at the genus level. Finally, our study is descriptive and does not include mechanistic experiments. The associations we report are intended as a foundational roadmap to guide future hypothesis‐driven research using in vivo and in vitro models to explore the functional consequences of these microbial changes.

## 5 Conclusion

In conclusion, our study provides the first evidence of significant gut dysbiosis in HTLV‐1 infection that correlates with disease progression to HAM/TSP. Although these observational results cannot prove causality, they do provide solid scientific conclusions about the relationship between the gut microbiome and HTLV‐1 pathology. These results open new avenues for research and form hypotheses for future diagnostic and therapeutic strategies, which, however, need to be developed considering the cross‐sectional design of the study and demographic confounding factors.

## Author Contributions

Lorena Abreu Fernandes, Youko Nukui, Rosa Maria Marcusso, Augusto César Penalva de Oliveira, Jorge Casseb, Patricia Bianca Clissa, and Sabri Saeed Sanabani collected samples and information. Lorena Abreu Fernandes, Ana Olivia de Souza, Patricia Bianca Clissa, Silas G. Villas‐Boas, and Sabri Saeed Sanabani performed data analysis and investigation. All authors revised the manuscript, contributed to the article, and approved the submitted version of the manuscript.

## Ethics Statement

This study was approved by the Institutional Review Board of the Emilio Ribas Institute (CAAE: 68008923.5.1001.0061) and the Hospital das Clínicas (CAAE: 65467022.7.0000.0068). All the participants provided informed consent to participate in the study.

## Conflicts of Interest

The authors declare no conflicts of interest.

## Supporting information


**Figure S1:** Composition and abundance of bacterial communities at the class Level. The plot illustrates the bacterial community composition and abundance across different groups: 38 patients with HAM, 17 with IS, 33 with ASC, and 13 representative samples from healthy controls (HCs). The sample sizes were adjusted to 100 to ensure clarity in the presentation.

**Figure S2:** Composition and abundance of bacterial communities at the order level. The plot illustrates the bacterial community composition and abundance across different groups: 38 patients with HAM, 17 with IS, 33 with ASC, and 13 representative samples from healthy controls (HCs). The sample sizes were adjusted to 100 to ensure clarity in the presentation.

**Figure S3:** Composition and abundance of bacterial communities at the family level. The plot illustrates the bacterial community composition and abundance across different groups: 38 patients with HAM, 17 with IS, 33 with ASC, and 13 representative samples from healthy controls (HCs). The sample sizes were adjusted to 100 to ensure clarity in the presentation.

**Figure S4:** Average composition and abundance of bacterial communities at the class level. The average composition and relative abundance of the bacterial communities of PLHTLV‐1 (*n* = 88) and HC (*n* = 24) at the class level in the recruited cohort.

**Figure S5:** Average composition and abundance of bacterial communities at the order level. The average composition and relative abundance of the bacterial communities of PLHTLV‐1 (*n* = 88) and HC (*n* = 24) at the order level in the recruited cohort.

**Figure S6:** Average composition and abundance of bacterial communities at the family level. The average composition and relative abundance of the bacterial communities of PLHTLV‐1 (*n* = 88) and HC (*n* = 24) at the family level in the recruited cohort.

**Figure S7:** Comparison of bacterial community composition at the class level. Comparison of the gut bacteriome composition between PLHTLV‐1 (*n* = 88) and HC (*n* = 24) at the class level.

**Figure S8:** Comparison of bacterial community composition at the order level. Comparison of the gut bacteriome composition between PLHTLV‐1 (*n* = 88) and HC (*n* = 24) at the order level.

**Figure S9:** Comparison of bacterial community composition at the family level. Comparison of the gut bacteriome composition between PLHTLV‐1 (*n* = 88) and HC (*n* = 24) at the family level.

**Figure S10:** Heatmap analysis of selected OTUs. The heatmap displays the relative abundance of 17 key OTUs (out of multiple OTUs analyzed) across PLHTLV‐1 (*n* = 88) and HC (*n* = 24) groups. While certain genera (e.g., *Faecalibacterium*, *Blautia*, *Bacteroides*) show differential abundance, the dendrogram (upper part) reveals mixed clustering of samples, indicating similar overall bacterial community structures between the two groups. This suggests that HTLV‐1 infection does not significantly alter the gut microbiome composition at the OTU level.

**Figure S11:** Multivariate analysis of microbial community structures. Beta‐diversity significance analysis was performed using EzBioCloud (q2‐diversity) with 999 permutations. Panels (A–F) visualize the results of the PERMANOVA analysis, comparing microbial community structures across groups.

## Data Availability

The datasets presented in this study can be found in the Zenodo repository with the following DOI https://doi.org/10.5281/zenodo.14679800 and https://doi.org/10.5281/zenodo.14674137.
