# Supplementary figures and images for: Gut Microbiome Dysbiosis is Associated With Human T‐Lymphotropic Virus Type 1 (HTLV‐1) Infection and Disease Progression to HTLV‐1‐Associated Myelopathy/Tropical Spastic Paraparesis: A Cross‐Sectional Study
Source: Smart Med. 2026 Jan 9;5(1):e70024. doi: 10.1002/smmd.70024 (PMC12794673; doi:10.1002/smmd.70024)

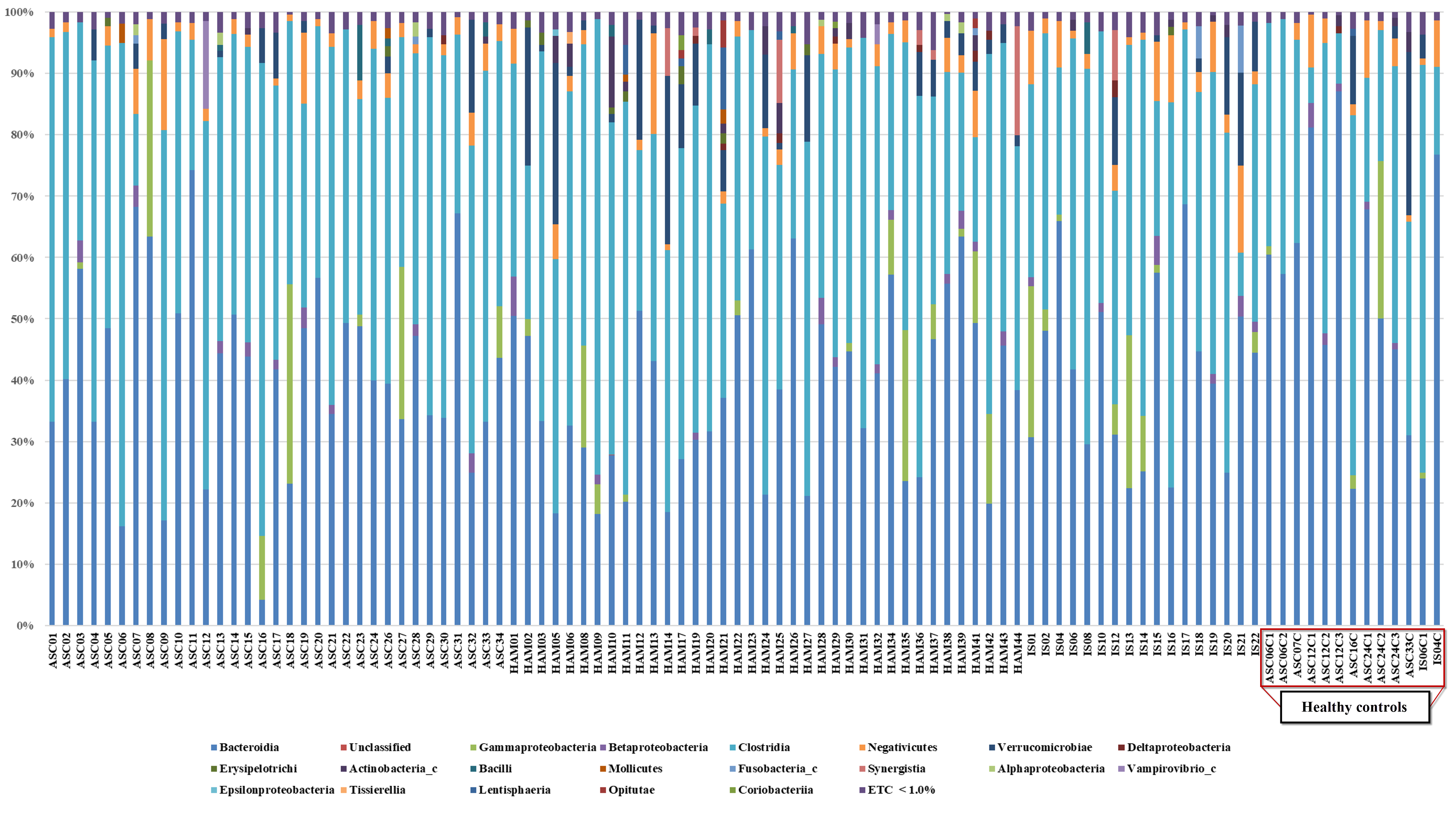

Supplement: Supplementary file 1 — Figure S1: Composition and abundance of bacterial communities at the class Level. The plot illustrates the bacterial community composition and abundance across different groups: 38 patients with HAM, 17 with IS, 33 with ASC, and 13 representative samples from healthy controls (HCs). The sample sizes were adjusted to 100 to ensure clarity in the presentation. [file SMMD-5-e70024-s008.tif]

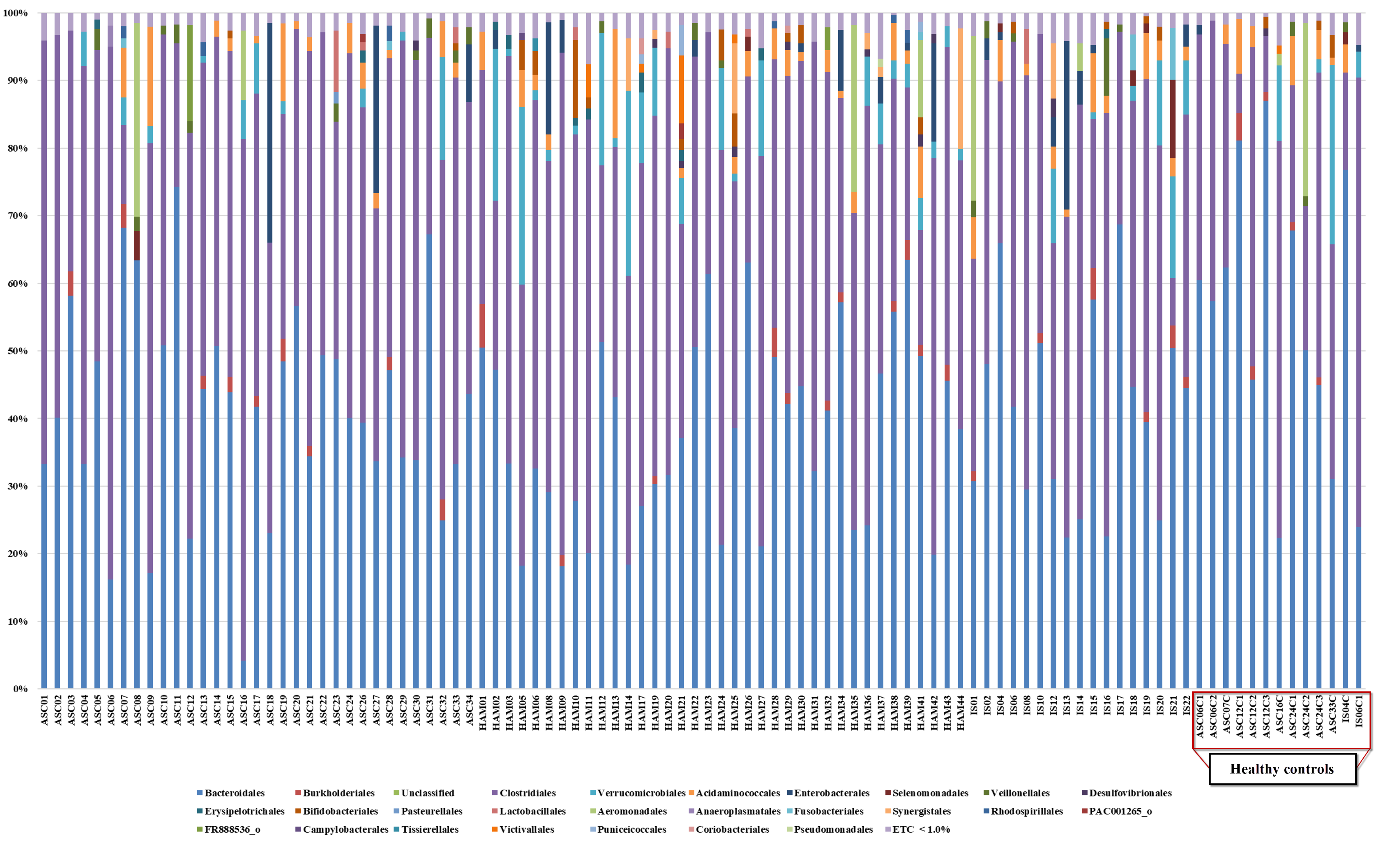

Supplement: Supplementary file 2 — Figure S2: Composition and abundance of bacterial communities at the order level. The plot illustrates the bacterial community composition and abundance across different groups: 38 patients with HAM, 17 with IS, 33 with ASC, and 13 representative samples from healthy controls (HCs). The sample sizes were adjusted to 100 to ensure clarity in the presentation. [file SMMD-5-e70024-s003.tif]

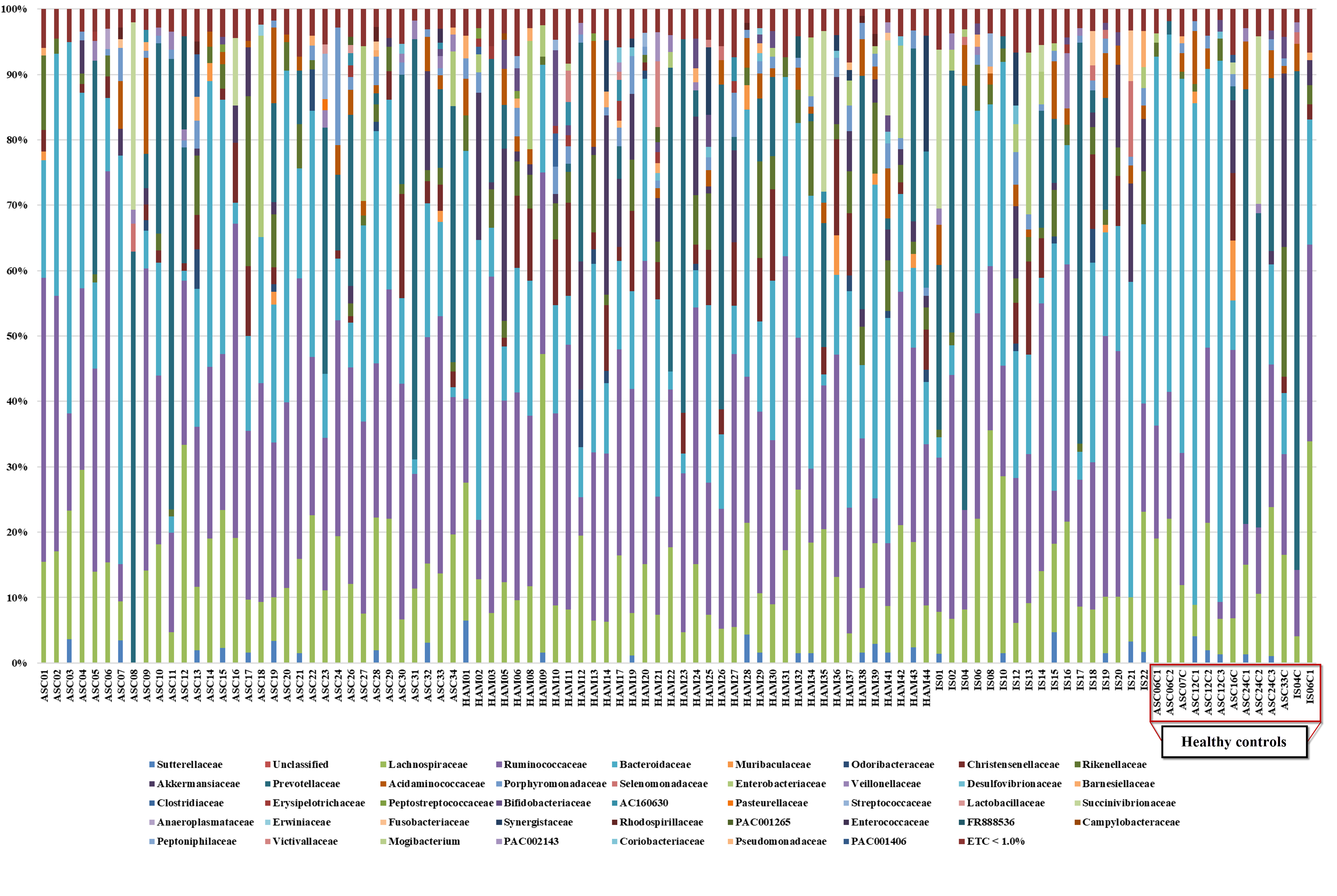

Supplement: Supplementary file 3 — Figure S3: Composition and abundance of bacterial communities at the family level. The plot illustrates the bacterial community composition and abundance across different groups: 38 patients with HAM, 17 with IS, 33 with ASC, and 13 representative samples from healthy controls (HCs). The sample sizes were adjusted to 100 to ensure clarity in the presentation. [file SMMD-5-e70024-s005.tif]

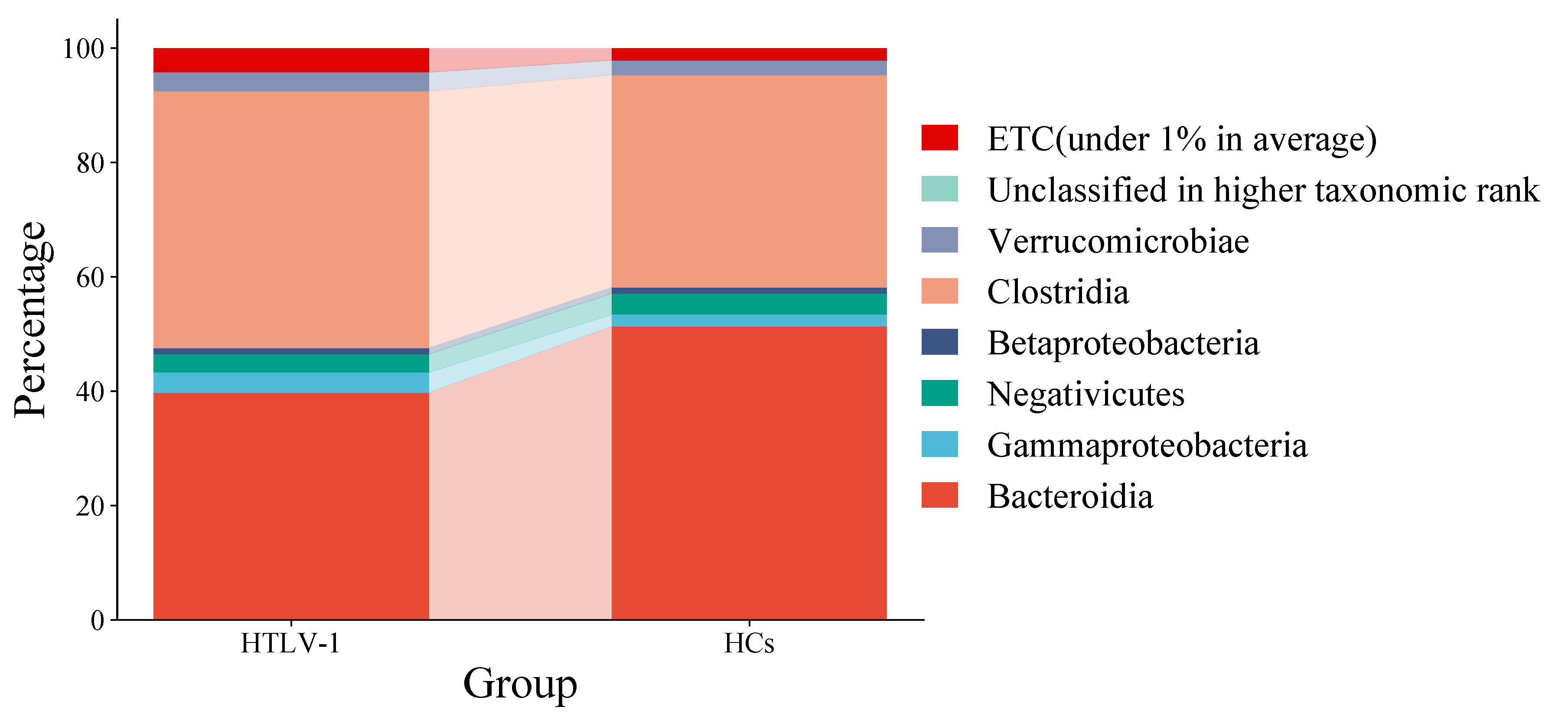

Supplement: Supplementary file 4 — Figure S4: Average composition and abundance of bacterial communities at the class level. The average composition and relative abundance of the bacterial communities of PLHTLV‐1 (n = 88) and HC (n = 24) at the class level in the recruited cohort. [file SMMD-5-e70024-s011.png]

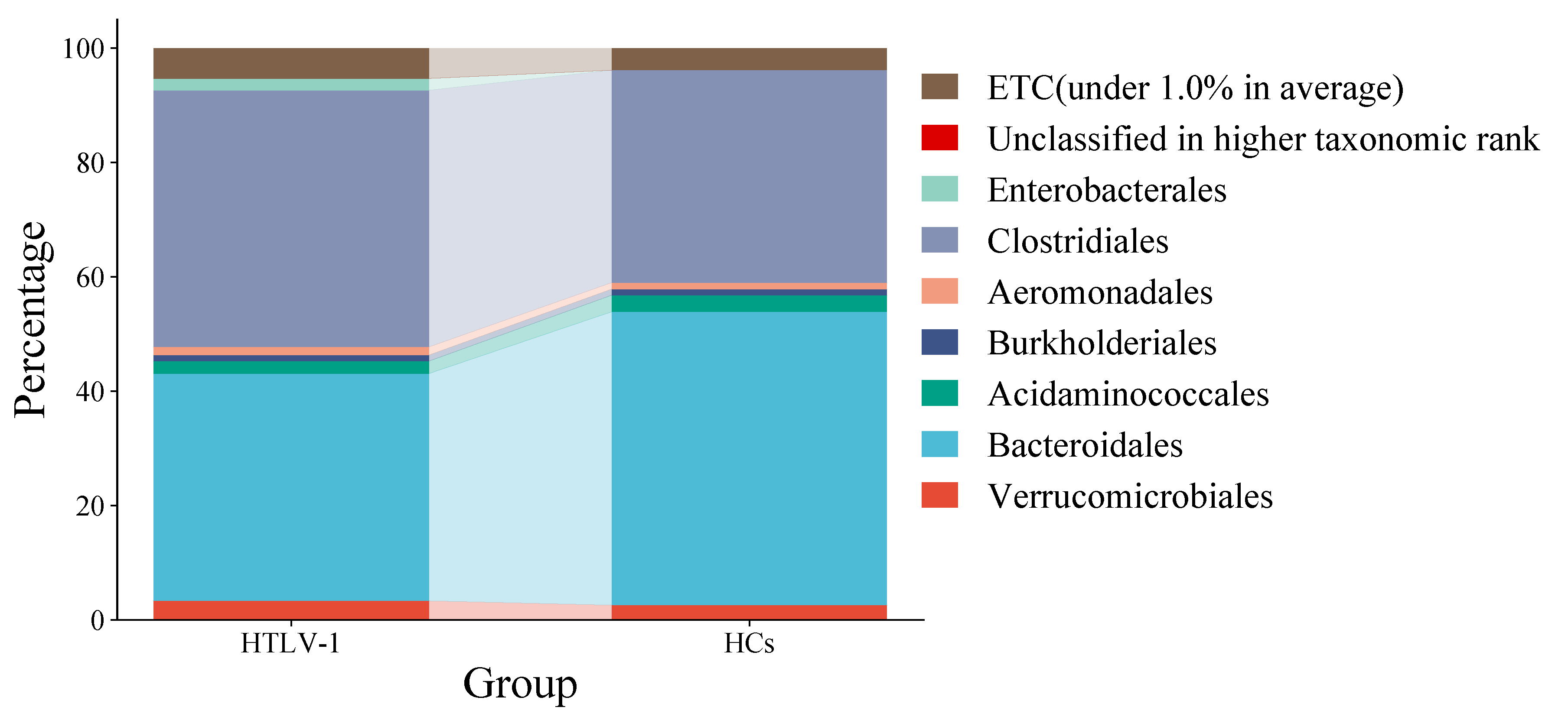

Supplement: Supplementary file 5 — Figure S5: Average composition and abundance of bacterial communities at the order level. The average composition and relative abundance of the bacterial communities of PLHTLV‐1 (n = 88) and HC (n = 24) at the order level in the recruited cohort. [file SMMD-5-e70024-s002.png]

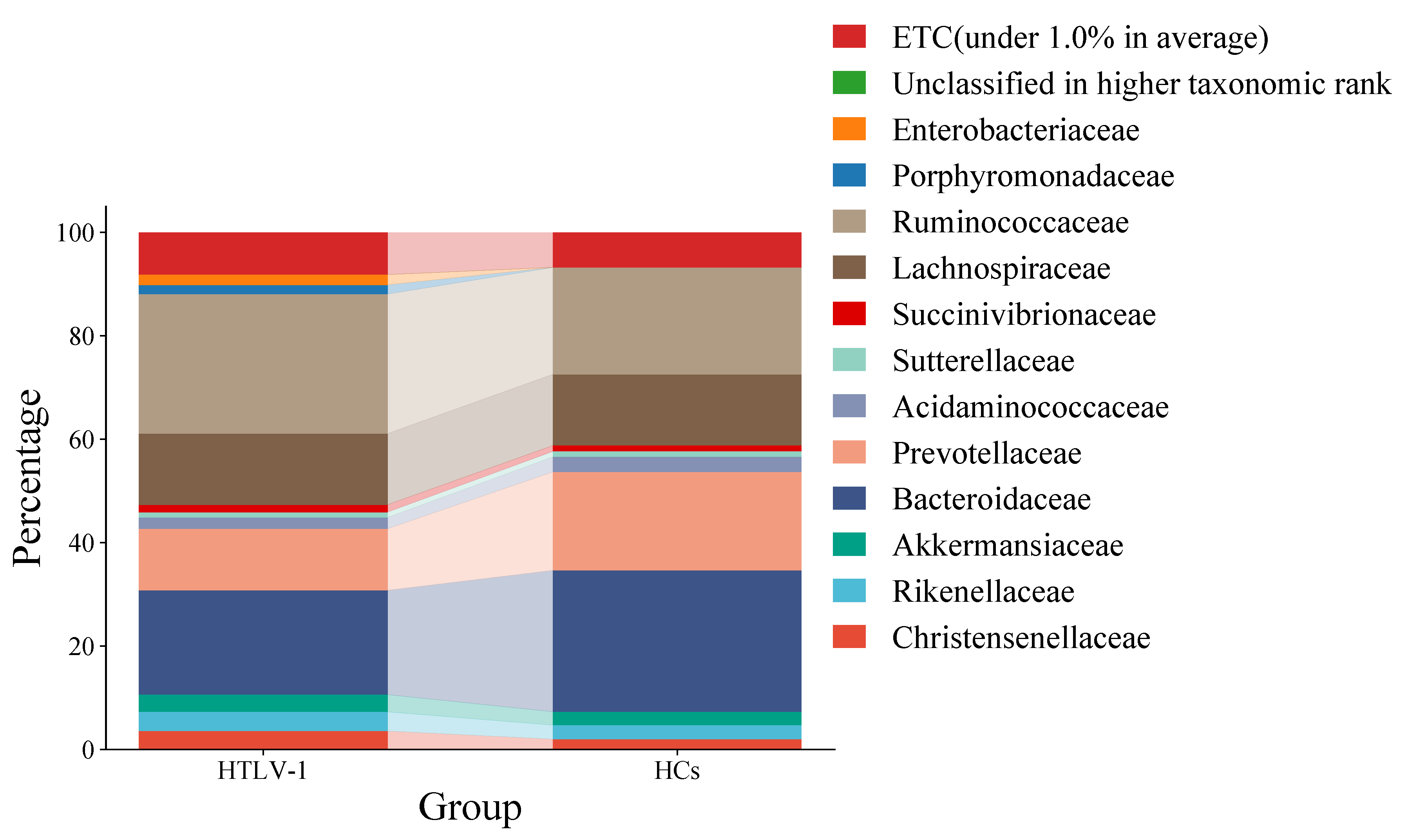

Supplement: Supplementary file 6 — Figure S6: Average composition and abundance of bacterial communities at the family level. The average composition and relative abundance of the bacterial communities of PLHTLV‐1 (n = 88) and HC (n = 24) at the family level in the recruited cohort. [file SMMD-5-e70024-s001.png]

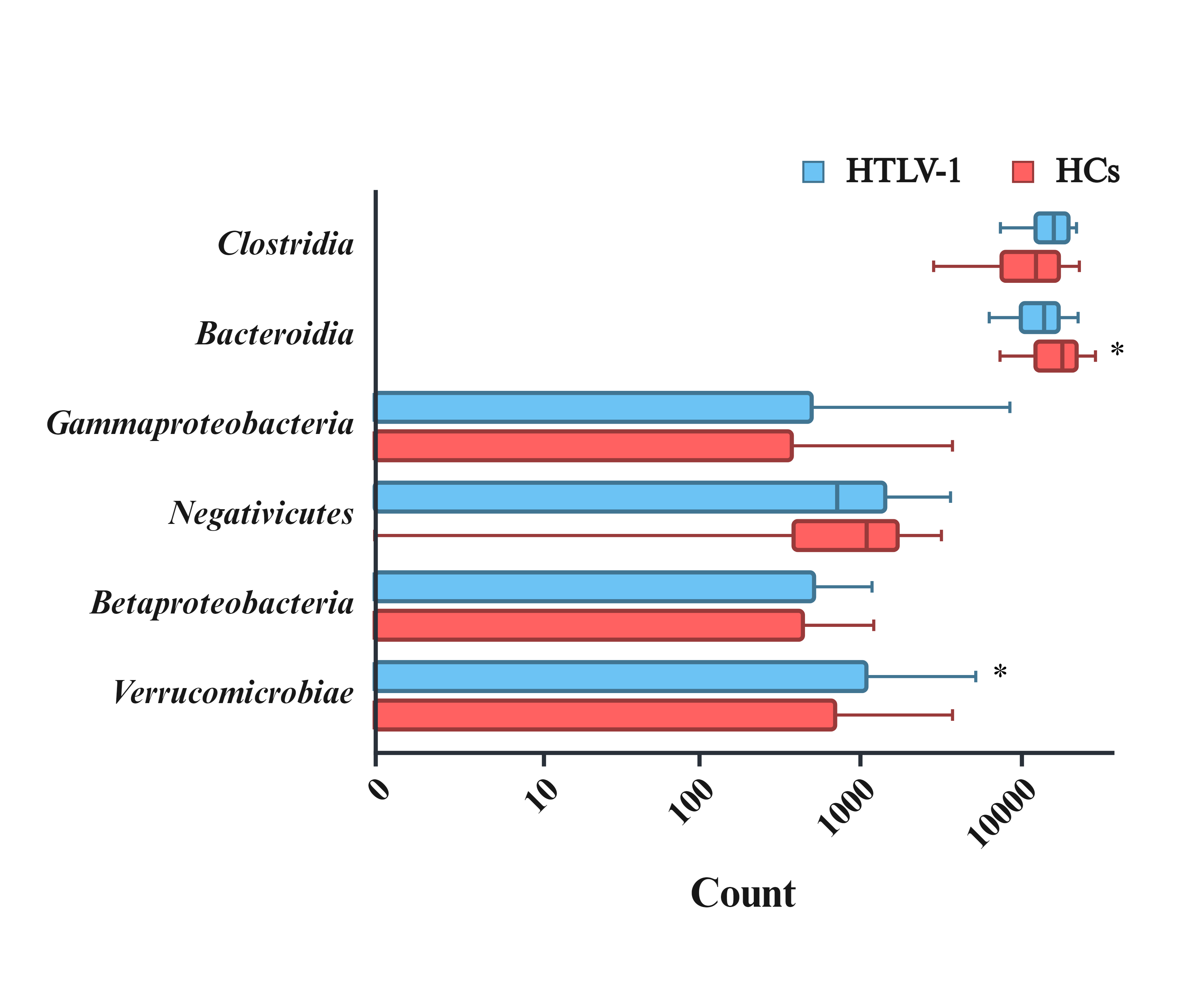

Supplement: Supplementary file 7 — Figure S7: Comparison of bacterial community composition at the class level. Comparison of the gut bacteriome composition between PLHTLV‐1 (n = 88) and HC (n = 24) at the class level. [file SMMD-5-e70024-s010.png]

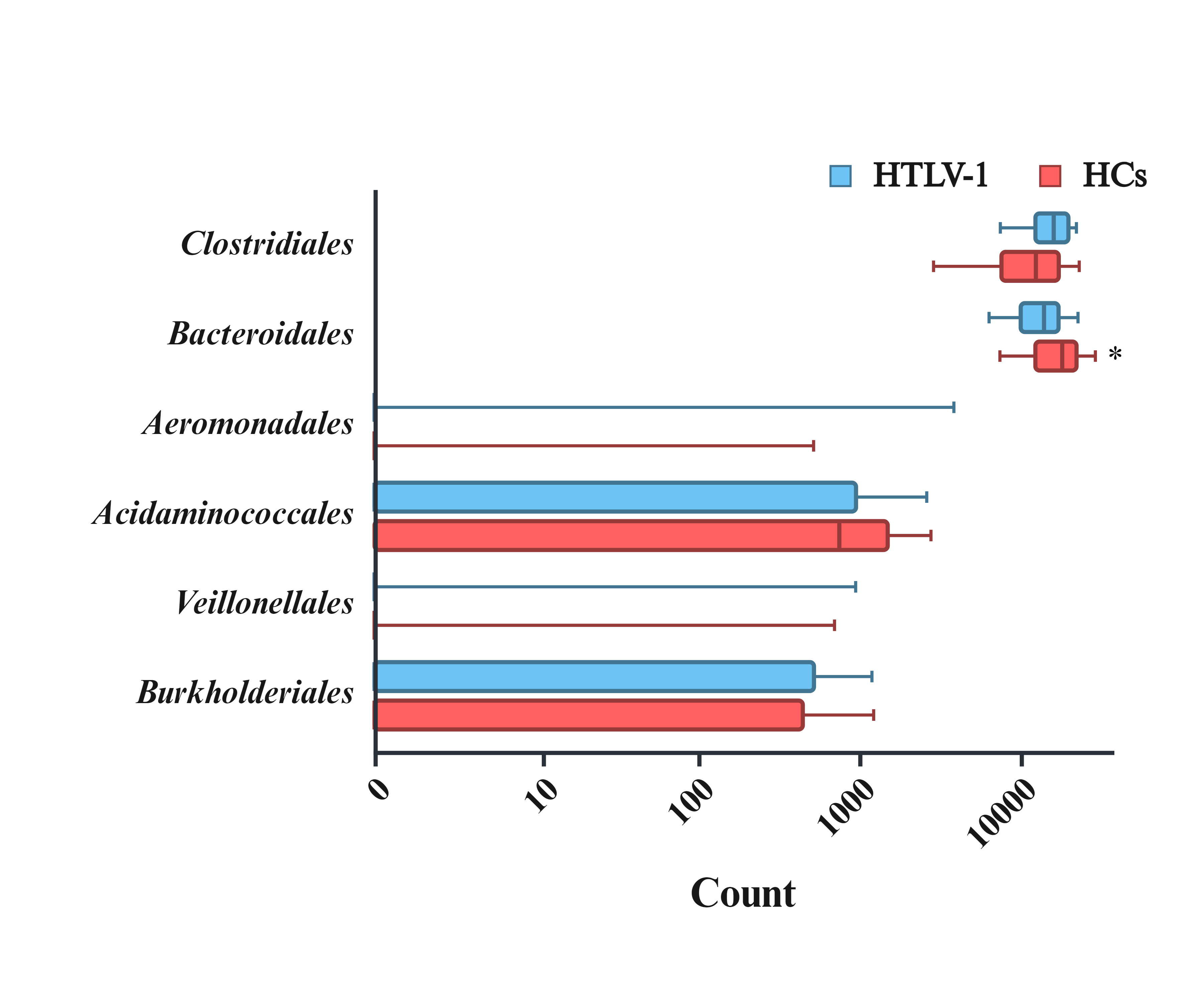

Supplement: Supplementary file 8 — Figure S8: Comparison of bacterial community composition at the order level. Comparison of the gut bacteriome composition between PLHTLV‐1 (n = 88) and HC (n = 24) at the order level. [file SMMD-5-e70024-s006.png]

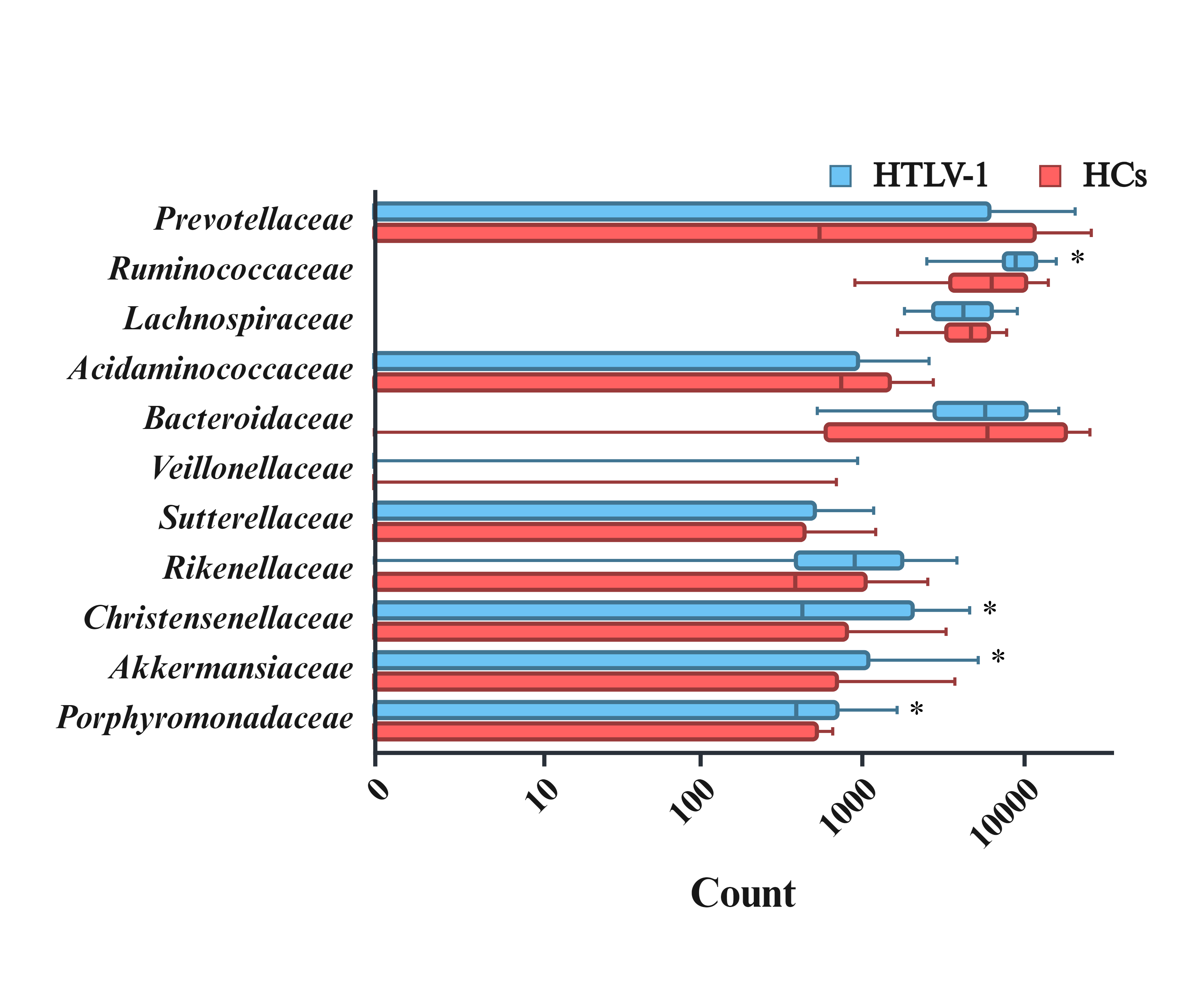

Supplement: Supplementary file 9 — Figure S9: Comparison of bacterial community composition at the family level. Comparison of the gut bacteriome composition between PLHTLV‐1 (n = 88) and HC (n = 24) at the family level. [file SMMD-5-e70024-s004.png]

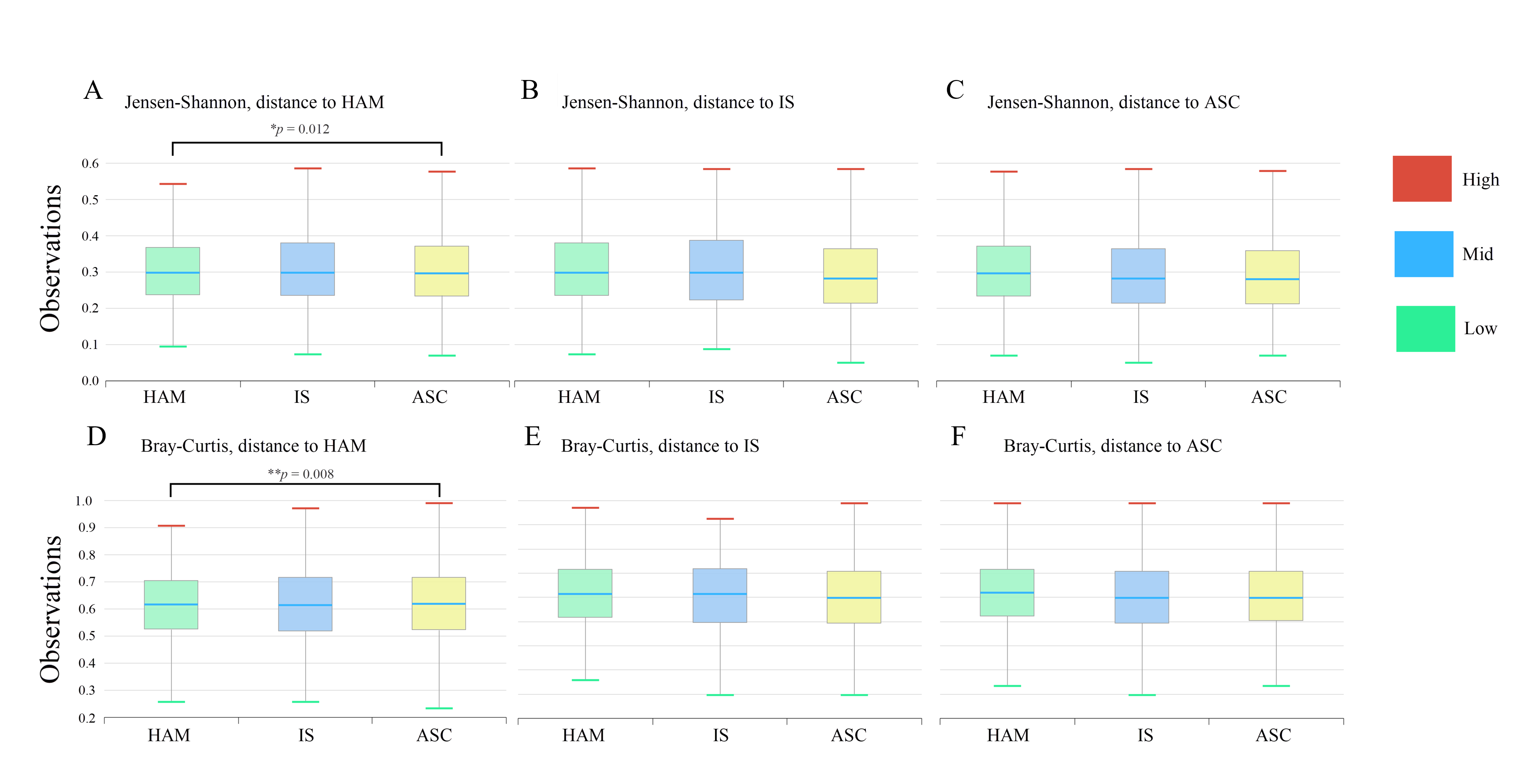

Supplement: Supplementary file 11 — Figure S11: Multivariate analysis of microbial community structures. Beta‐diversity significance analysis was performed using EzBioCloud (q2‐diversity) with 999 permutations. Panels (A–F) visualize the results of the PERMANOVA analysis, comparing microbial community structures across groups. [file SMMD-5-e70024-s009.png]
